# Supplementary figures and images for: Structural and biochemical characterization of the key components of an auxin degradation operon from the rhizosphere bacterium Variovorax
Source: PLoS Biol. 2023 Jul 17;21(7):e3002189. doi: 10.1371/journal.pbio.3002189 (PMC10374108; doi:10.1371/journal.pbio.3002189)

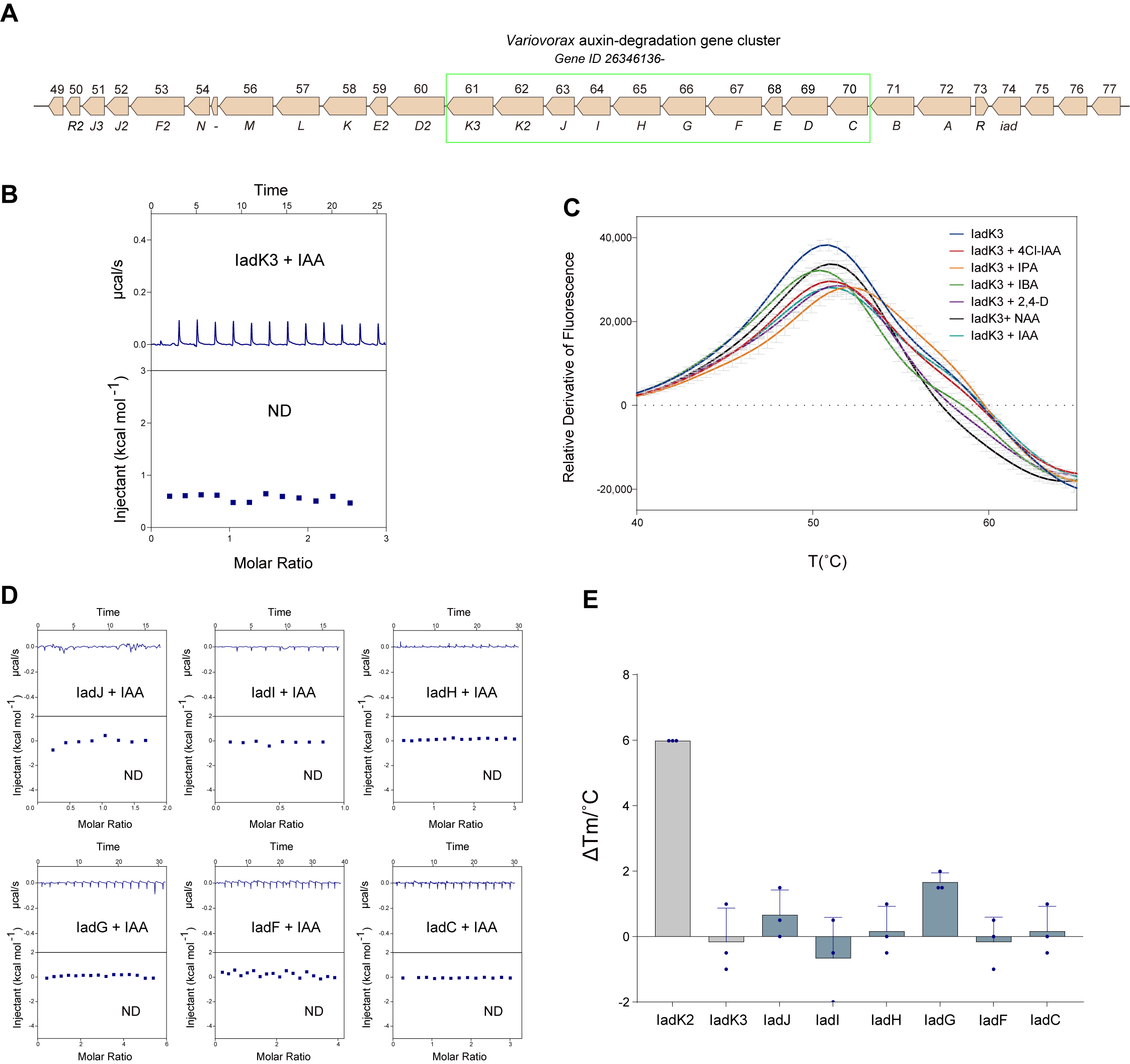

Supplement: S1 Fig — (A) Schematics for the auxin IAA-degradation (iad) operon in Variovorax paradoxus CL14. The green box highlights the 10 genes examined in the study for IAA transformation. (B) ITC measurement of IAA binding to iadk3. “ND” indicates no binding detected. (C) Thermal shift assay for iadk3 with IAA and different analogs including 4Cl-IAA, IPA, IBA, 2,4-D, and NAA. Three replicates of each TSA experiments were performed. (D) Integrated heat plots for ITC measurements of different Iad proteins binding with IAA. Measurements for proteins iadf-J and iadc with IAA are displayed. ND: no binding detected. Three or more independent measurements were performed. (E) Tm changes for Iad proteins in the presence of IAA in the thermal shift assay. Three replications of each TSA experiments were performed. While adding of IAA substantially increased the melting temperature of iadk2, no profound effects were observed for the rest. Source data for B–E can be found in S1 Data. (TIF) [file pbio.3002189.s001.tif]

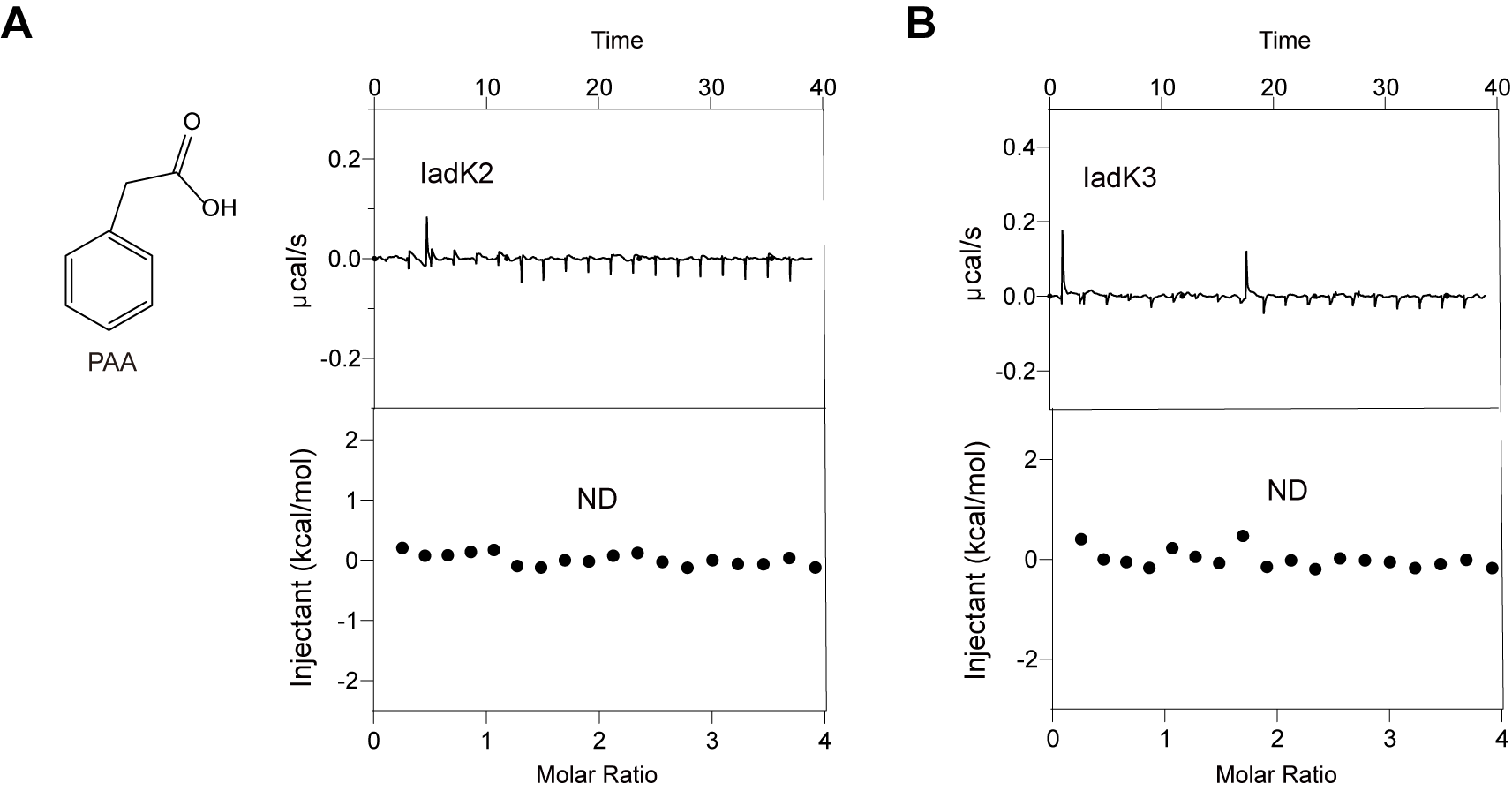

Supplement: S2 Fig — (A) Integrated heat plots for ITC measurement of iadk2 binding with PAA. ND: no binding detected. (B) Integrated heat plots for ITC measurements of IadK3 binding with PAA. ND: no binding detected. Two or more measurements were performed. Source data can be found in S1 Data. (TIF) [file pbio.3002189.s002.tif]

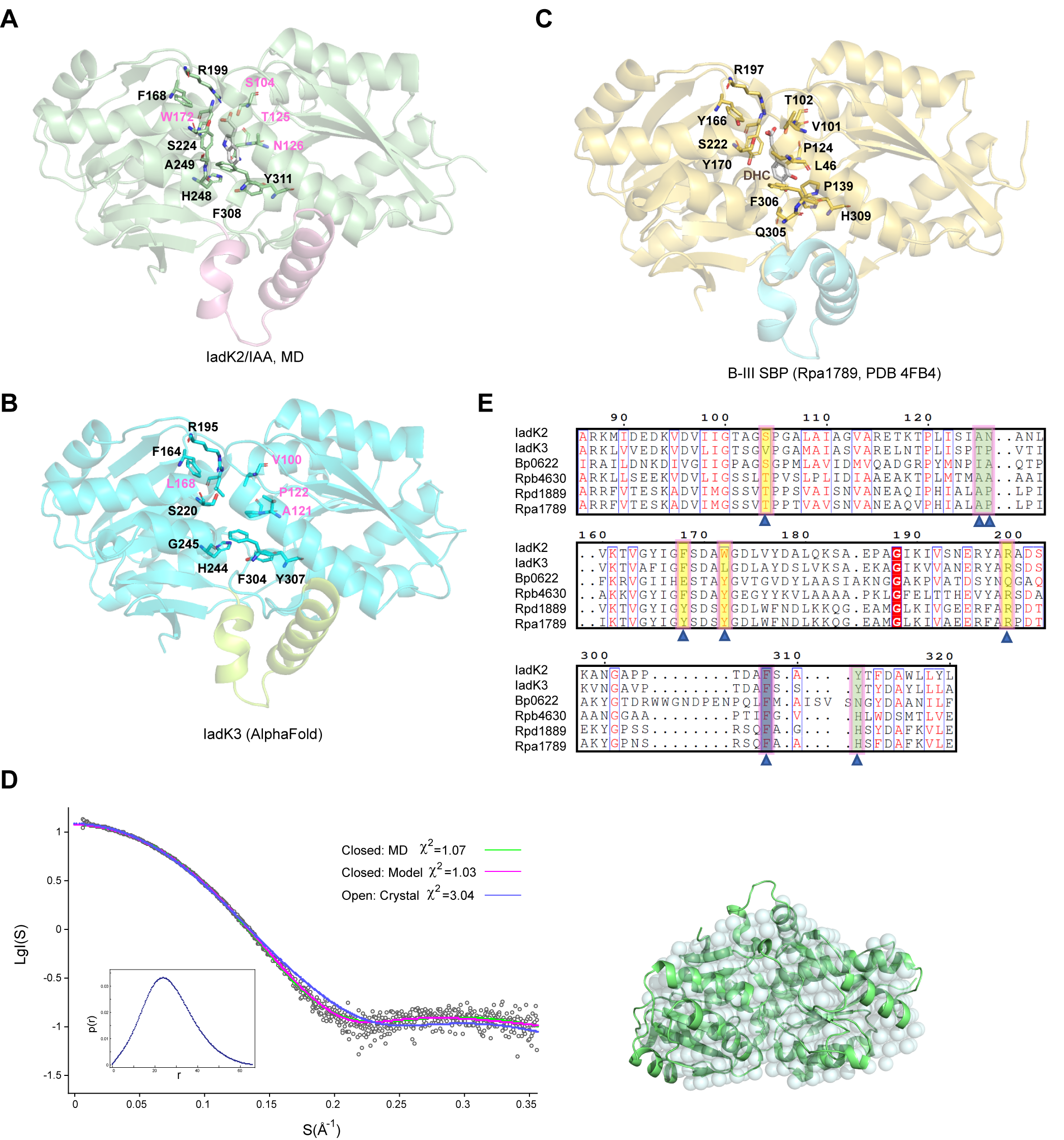

Supplement: S3 Fig — (A) The overall structure and the calculated IAA-binding pocket of iadk2 using docking and MD simulation. The L2 linker is colored in pink and the rest is in pale green. Key residues interacting with IAA are shown in sticks representation. (B) Structure of iadk3 predicted using alphafold2. The L2 linker is colored in green and the rest is in cyan. The counter residues of iadk2 involved in IAA binding iniadk3 are indicated. Residues labeled in magenta are not conserved between iadk3 and iadk2, which could be related to the IAA specificity of iadk2. (C) The closest structural homolog of iadk2 according to the Dali search. The structure of an SBP from Rhodopseudomonas palustris in complex with caffeic acid (DHC) is displayed (PDB: 4FB4). The structure containing 2 α helices in L2 belongs to the B-III subcluster of sbps. Key residues involved in substrate DHC binding are shown. (D) SAXS analysis of iadk2 complexed with IAA. Left panel: overlaid scattering pattern of the SAXS ab initio model (pink line), the theoretical scattering profiles of iadk2-IAA complex structure from MD simulation (green line) and iadk2 apo crystal structure (blue line) with SAXS scattering data of iadk2-IAA complex. The inserted panel displays the P(r) distance distributions. Right: Superposition of the ab initio SAXS model (shown as sphere) and the iadk2-IAA complex structure from MD simulation. Source data for D can be found in S1 Data. (E) Multiple sequence alignment. Sequences of iadk2, iadk3, and B-III sbps including Bp0622 (Uniprot Q7VS30), Rpb4630 (Uniprot Q2IR47), Rpd1889 (Uniprot Q139W5), and Rpa1789 (Uniprot Q6N8W4) were aligned with Clustal Omega. Essential residues for iadk2 IAA binding are marked with the filled triangles, among which the strictly conserved residues were shaded in blue, whereas moderately and less conserved residues were in yellow and light green, respectively. (TIF) [file pbio.3002189.s003.tif]

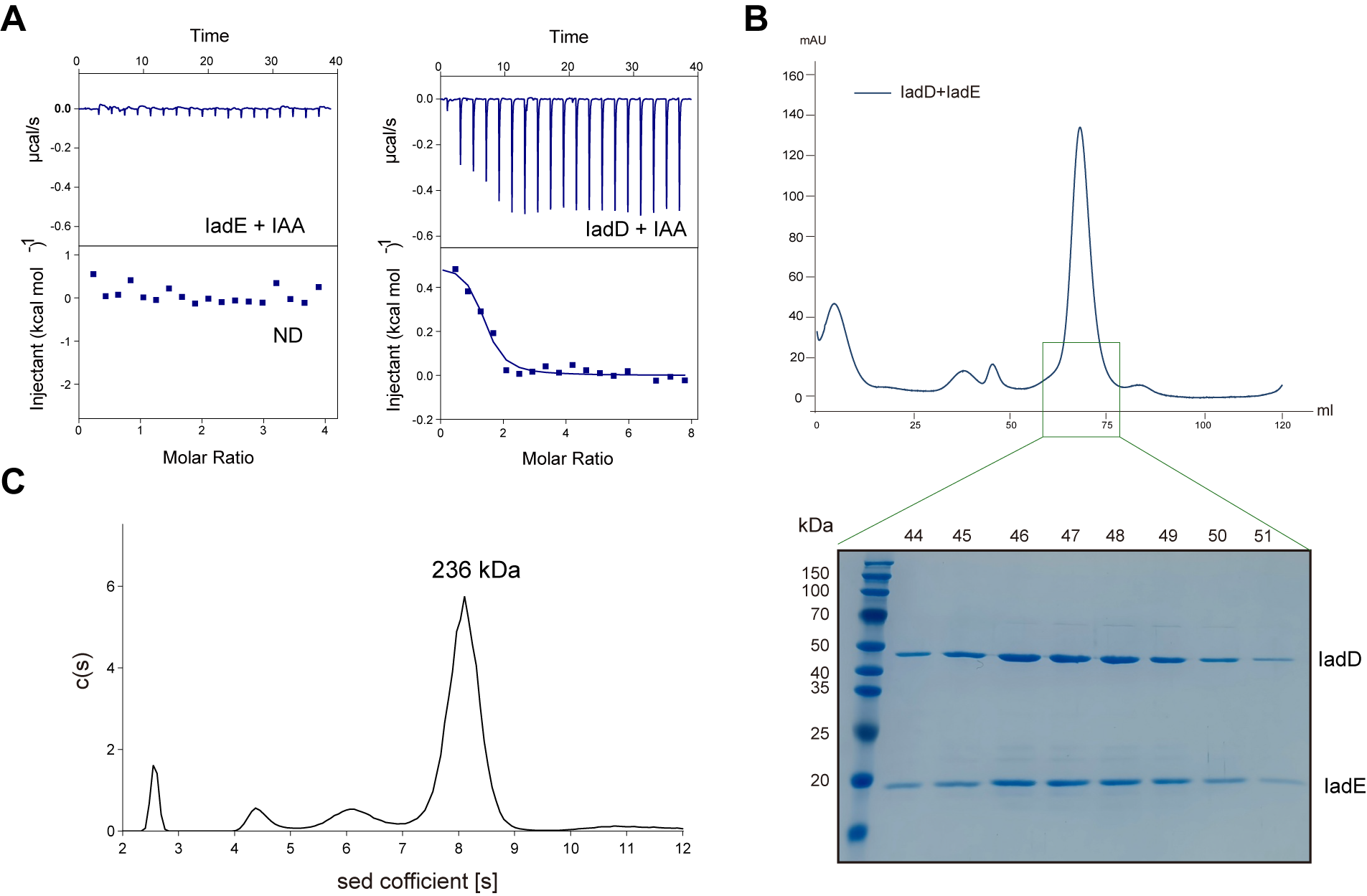

Supplement: S4 Fig — (A) ITC measurements of IAA binding to iadd or iade protein alone. ND: no binding detected. Left panel: no binding between iade and IAA was detected. Right panel: a fitted Kd value of 24 μM was obtained for iadd and IAA. Source data for A can be found in S1 Data. (B) Size-exclusion chromatograms of iadd and iade. The elution fractions from the complex peak were analyzed by SDS-PAGE gel (lower panel). Uncropped SDS-PAGE gel image can be found in S1 Raw Image. (C) Sedimentation coefficient distributions of iadd/E complex. (TIF) [file pbio.3002189.s004.tif]

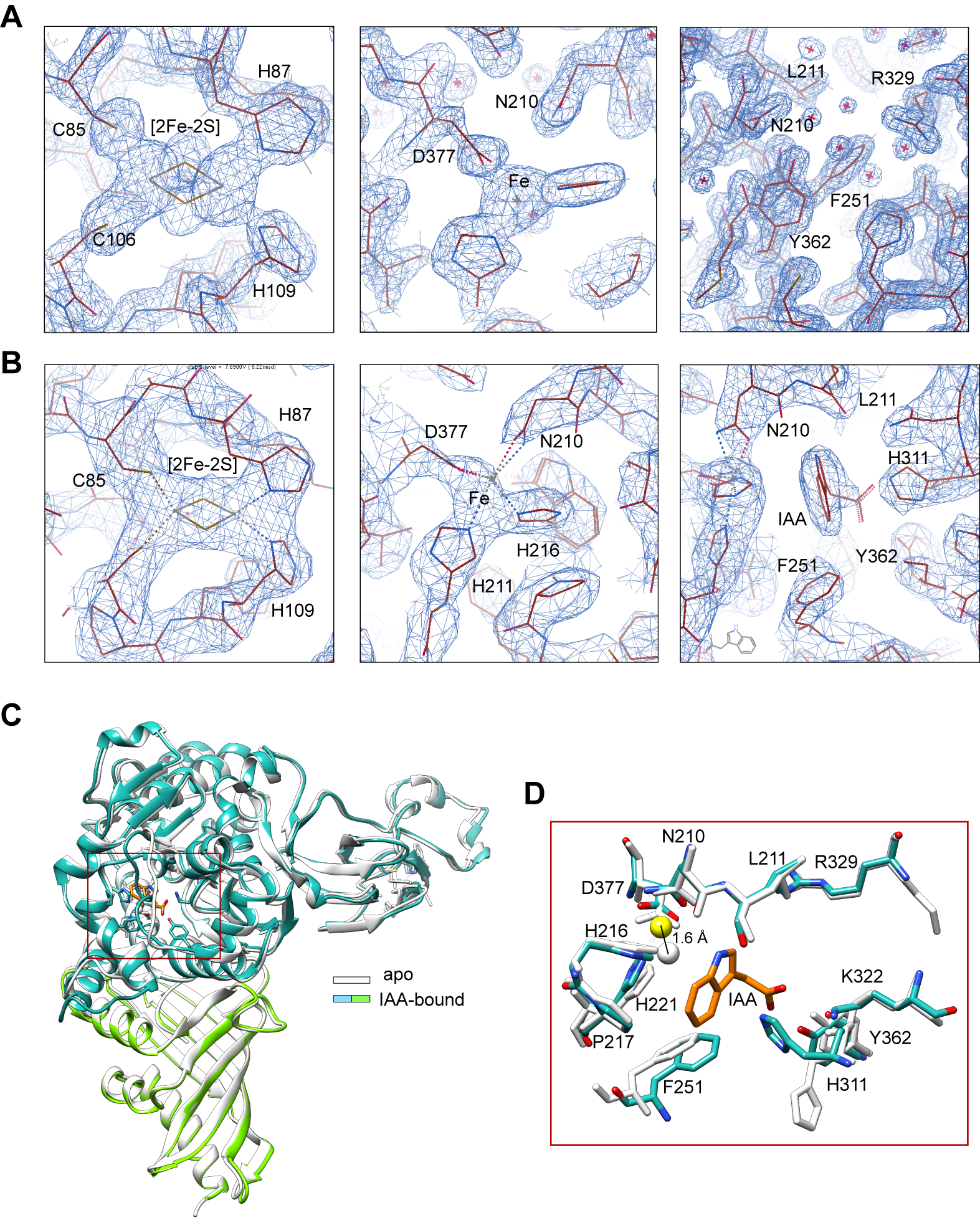

Supplement: S5 Fig — (A) Electron densities and the atomic model around the active site of apo iadd/E. Densities and models around [2Fe-2S], Fe and the substrate-binding pocket were shown. (B) Electron densities and the atomic model around the active site of IAA-bound iadd/E. (C) Structural comparison of the apo and IAA-bound iadd/E structures. For clarity, 1 heterodimer subunit is displayed. The apo structure is colored in white and IAA-bound structure is colored by proteins. No profound overall conformational changes were identified between the apo and the IAA-bound structures. (D) A zoom in view of c around the active site. The side chain of H311 in IadD is relocated to accommodate the binding of IAA. Fe and coordinating residues also move slightly inwards (about 1.6 Å) following the binding of IAA. (TIF) [file pbio.3002189.s005.tif]

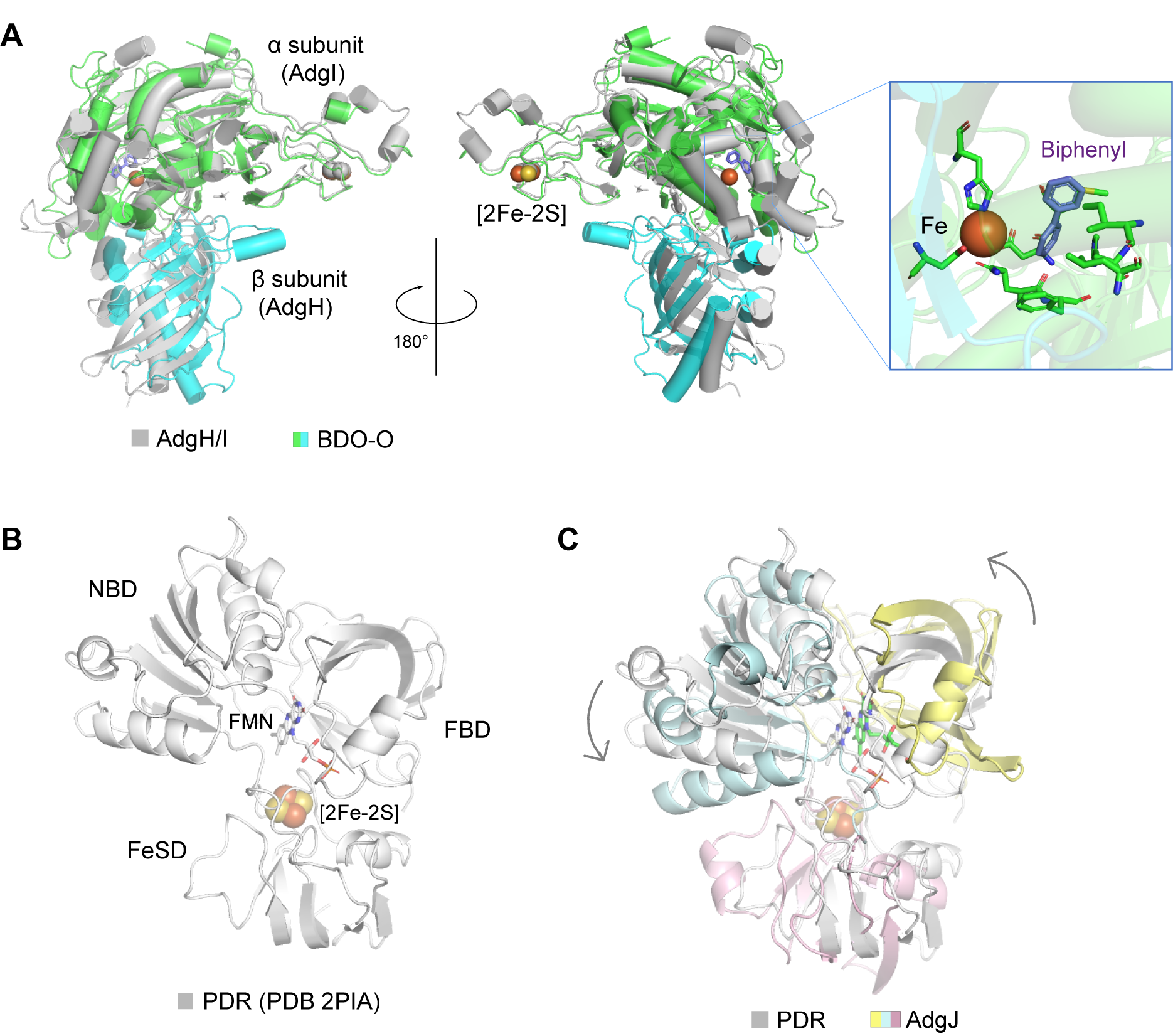

Supplement: S6 Fig — (A) Structural comparison of iadd/E-IAA with the homolog structure (PDB: 5AEW). For clarity, a single heterodimer is displayed. Overall, the 2 structures superimposed well with each other, despite some difference in the relative orientation of the α and β subunits. The inset panel in the right shows the detailed insight into the superimposed substrate-binding pocket. (B) The homolog structure of iadc (PDR, PDB: 2PIA). The structure of PDR is also composed of 3 major domains, NBD, FBD, and fesd. Ligands FMN and the [2Fe-2S] cluster are indicated. (C) Structural comparison of IadC with PDR. PDR is colored in white and IadC is colored by domains. The arrows indicate the relative domain rotations of IadC in comparison to PDR. (TIF) [file pbio.3002189.s006.tif]

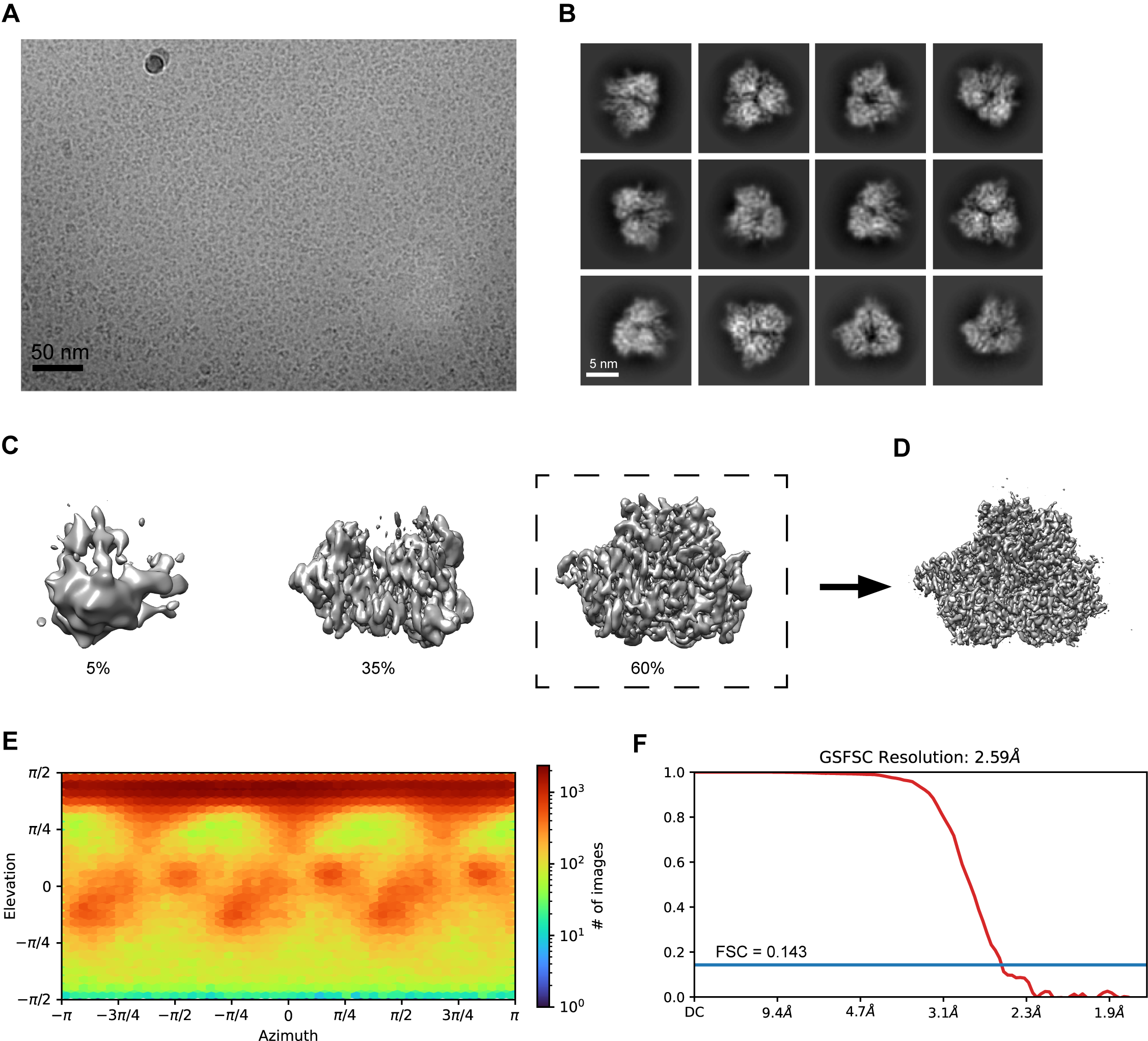

Supplement: S7 Fig — (A) A representative raw cryo-EM micrograph. Scale bar: 50 nm. (B) Representative 2D class averages. (C) 3D classification. Class 1 represent the bad reconstruction. Class 2 represents the broken protein complex. Class 3 represents the intact protein complex. (D) The final cryo-EM map of 3D refinement on Class 3. (E) Viewing distribution of the 3D refinement. (F) The Fourier shell correlation (FSC) curve of the reconstruction. The 0.143 gold standard FSC cutoff was used to determine the final resolution. (TIF) [file pbio.3002189.s007.tif]

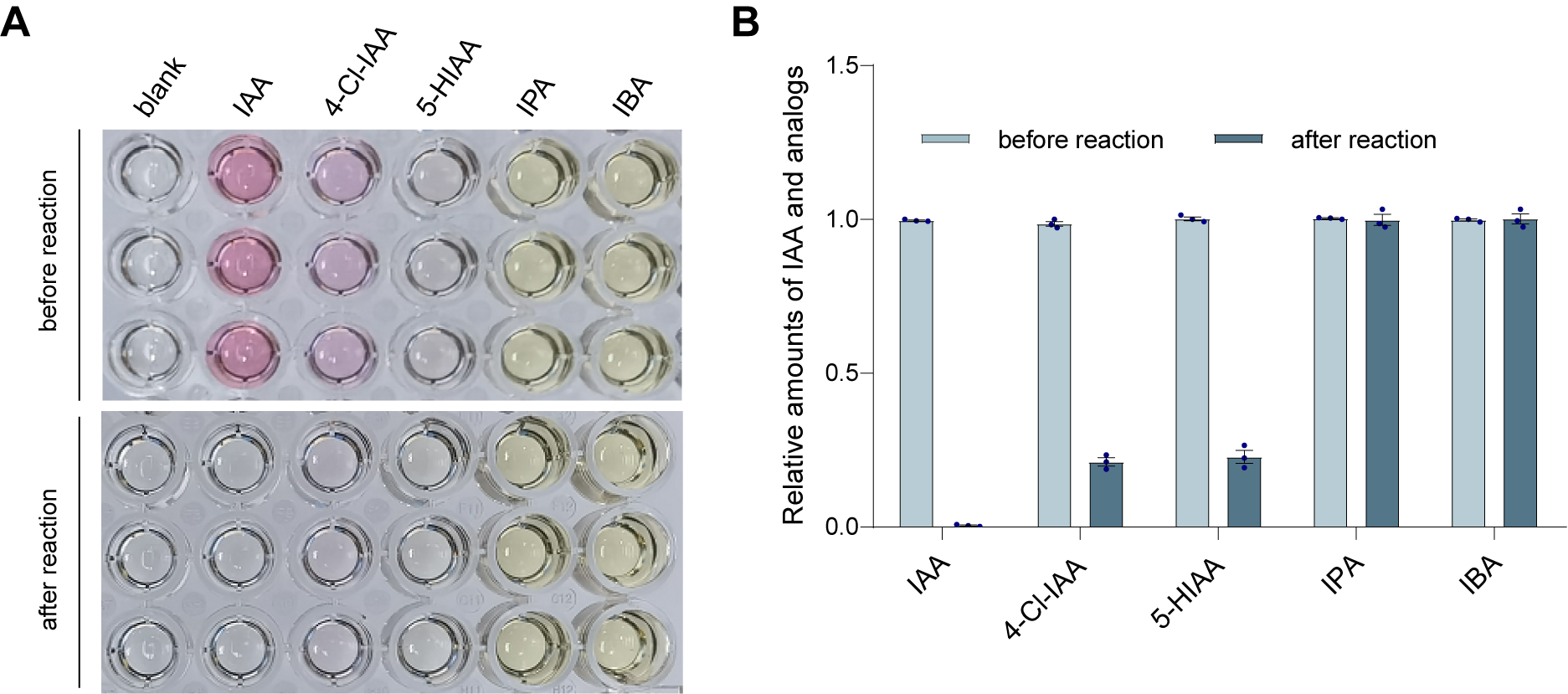

Supplement: S8 Fig — (A) Color development of IAA and analogs with the Salkowski reagent before and after treatments with iadc-iadd/E. (B) Quantification of the relative amounts of IAA or analogs before and after the oxidation reaction catalyzed by the iadc-iadd/E system. Three replications of each condition were performed. Data are presented as mean ± SEM. Source data for B can be found in S1 Data. (TIF) [file pbio.3002189.s008.tif]

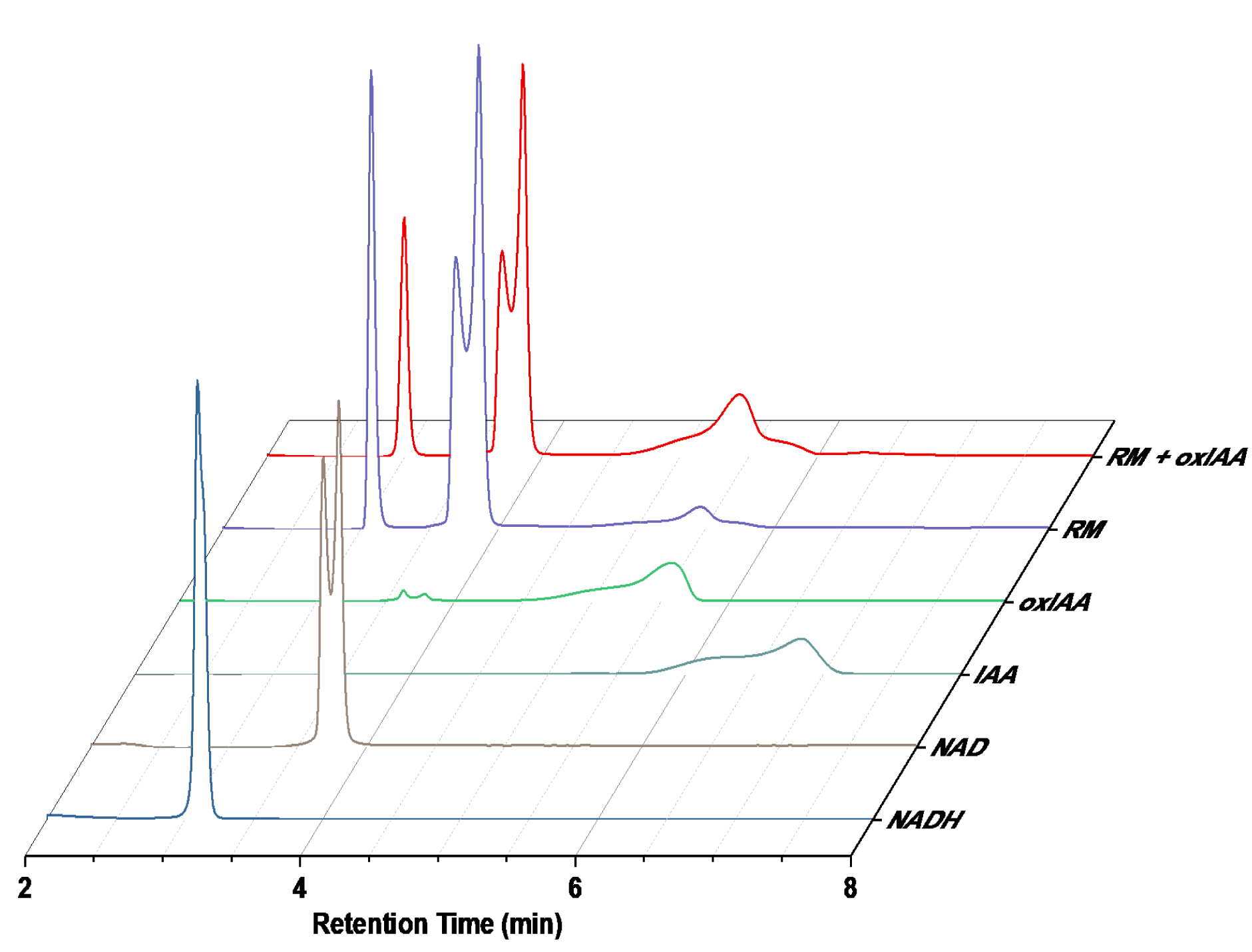

Supplement: S9 Fig — The reacted mixture (RM) was analyzed with HPLC. Analyses for NADH, NAD, IAA, and oxIAA were also performed to assign the peaks. (TIF) [file pbio.3002189.s009.tif]

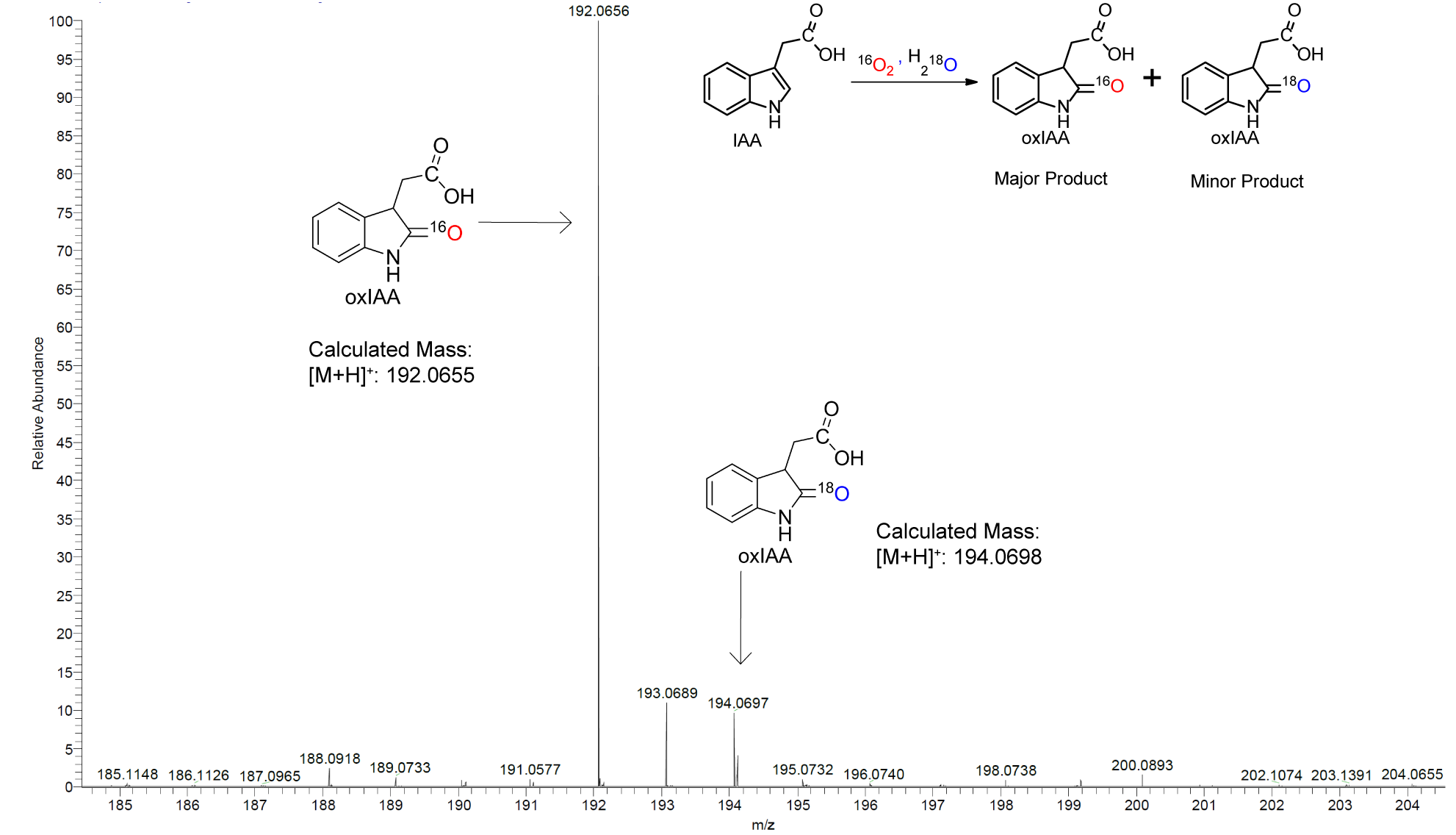

Supplement: S10 Fig — The in vitro IAA degradation reaction with IadC-IadD/E was carried out in the solution containing 95% H2O18 (v/v). (TIF) [file pbio.3002189.s010.tif]

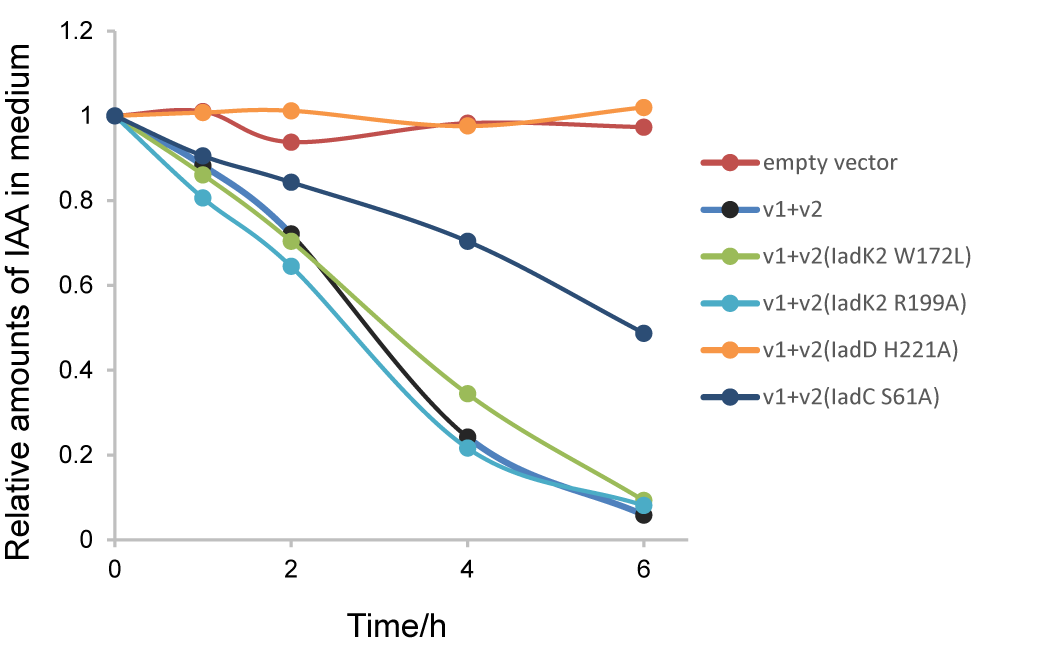

Supplement: S11 Fig — Effects of Iad proteins mutants in the in vivo IAA transformation by E. coli. Two vectors, v1 (containing genes IadK2 and IadC) and v2 (with genes IadD and IadE), were transformed to E. coli BL21(DE3) to enable IAA transformation. Mutants of IadK2 (W172L, R199A), IadD (H221A), and IadC (S61A) were tested. Little IAA conversion was observed with the empty vector without the iad genes or with the loss-of-function mutations in iadC or iadD. Source data can be found in S1 Data. (TIF) [file pbio.3002189.s011.tif]

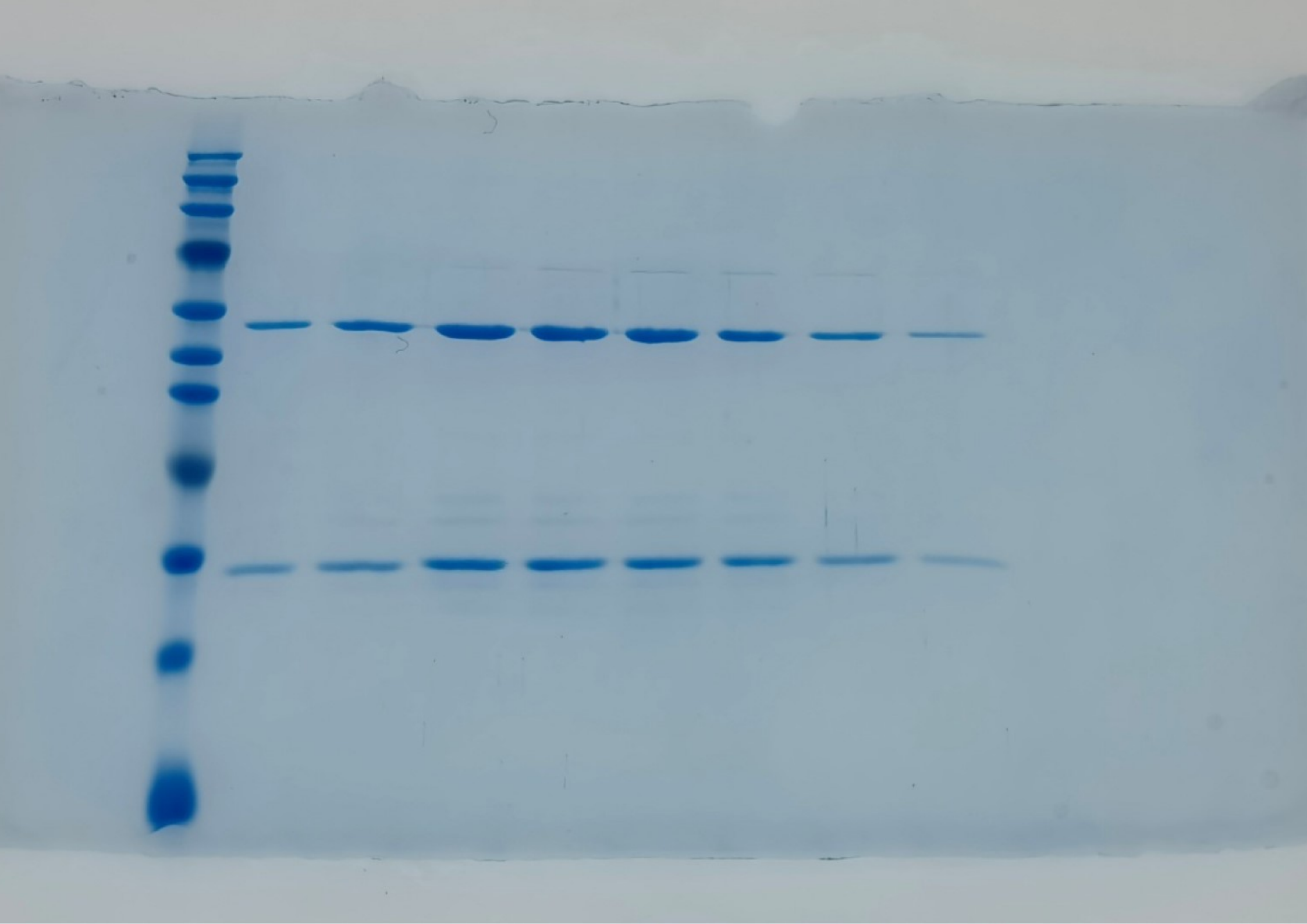

Supplement: S1 Raw Image — (PDF) [file pbio.3002189.s015.pdf]
