# Supplementary material for: Structural and biochemical characterization of the key components of an auxin degradation operon from the rhizosphere bacterium Variovorax
Source: PLoS Biol. 2023 Jul 17;21(7):e3002189. doi: 10.1371/journal.pbio.3002189 (PMC10374108; doi:10.1371/journal.pbio.3002189)
Supplement: S1 Table — (DOCX) [file pbio.3002189.s012.docx]

**S1 Table. Crystallographic data collection and refinement statistics**

|  | IadK2  (PDB ID:7YLT) | IadD/IadE  (PDB ID:7YLS) | IadC  (PDB ID:7YLR) |
| --- | --- | --- | --- |
| **Data collection** |  |  |  |
| Space group | P1 | P 23 | P 21 21 21 |
| Cell dimensions |  |  |  |
| *a*, *b*, *c* (Å) | 36.775 75.745 119.706 | 135.613 135.613 135.613 | 47.217 84.042 87.864 |
| α, β, γ (°) | 85.604 89.159 89.368 | 90 90 90 | 90 90 90 |
| Resolution (Å) | 48.67-2.3 (2.382-2.3) | 33.9-1.8 (1.864 -1.8) | 32.16-1.68 (1.74- 1.68) |
| *R*_sym_ or *R*_merge_ | 0.05453 (0.1948) | 0.1247 (0.7457) | 0.08067 (0.5343) |
| *I* / σ*I* | 9.78 (4.84) | 17.93 (2.67) | 18.40 (2.48) |
| Completeness (%) | 90.99 (83.71) | 99.97 (100.00) | 91.80 (59.32) |
| Redundancy | 3.6 (3.6) | 26.8 (13.1) | 11.1 (5.6) |
| CC1/2 | 0.998 (0.968) | 0.999 (0.884) | 0.999 (0.86) |
| **Refinement** |  |  |  |
| Resolution (Å) | 48.67-2.3 | 33.9-1.8 | 32.16-1.68 |
| No. reflections | 52101 | 76921 | 37285 |
| *R*_work_ / *R*_free_ | 0.1864/0.2276 | 0.1517/0.1683 | 0.1776/0.2087 |
| No. atoms | 11259 | 5279 | 2902 |
| Protein | 10737 | 4784 | 2467 |
| Ligand/ion | 0 | 40 | 35 |
| Water | 522 | 472 | 400 |
| *B*-factors | 34.07 | 33.88 | 25.58 |
| Protein | 34.09 | 33.12 | 24.51 |
| Ligand/ion |  | 42.10 | 16.68 |
| Water | 33.54 | 41.16 | 32.94 |
| R.m.s. deviations |  |  |  |
| Bond lengths (Å) | 0.003 | 0.013 | 0.007 |
| Bond angles (°) | 0.64 | 1.25 | 0.97 |
| Ramachandran  Favored (%)  Allowed (%)  Outliers (%) | 96.23  3.56  0.21 | 98.14  1.86  0.00 | 97.52  2.48  0.00 |
