# Supplementary material for: Structural and biochemical characterization of the key components of an auxin degradation operon from the rhizosphere bacterium Variovorax
Source: PLoS Biol. 2023 Jul 17;21(7):e3002189. doi: 10.1371/journal.pbio.3002189 (PMC10374108; doi:10.1371/journal.pbio.3002189)
Supplement: S2 Table — (DOCX) [file pbio.3002189.s013.docx]

**S2 Table. Cryo-EM data collection, refinement and validation statistics**

|  | IadD/IadE-IAA  (EMD-34443  PDB: 8H2T) |
| --- | --- |
| **Data collection and processing** |  |
| Magnification | 105,000 |
| Voltage (kV) | 300 |
| Electron exposure (e^–^/Å^2^) | 54 |
| Defocus range (μm) | -1.2 to -2.2 |
| Pixel size (Å) | 0.85 |
| Symmetry imposed | *C*3 |
| Initial particle images (no.) | 2,449,056 |
| Final particle images (no.) | 1,000,245 |
| Map resolution (Å) | 2.59 |
| FSC threshold | 0.143 |
| Map resolution range (Å) | 2.4 – 3.5 |
|  |  |
| **Refinement** |  |
| Initial model used | 7YLS |
| Model resolution (Å) | 3.0 |
| FSC threshold | 0.5 |
| Model resolution range (Å) | 2.6 – 3.1 |
| Map sharpening *B* factor (Å^2^) | -97 |
| Model composition |  |
| Non-hydrogen atoms | 14364 |
| Protein residues | 1776 |
| Ligands | 3 (FE)  3 (FES)  3 (IAC) |
| *B* factors (Å^2^) |  |
| Protein | 96.46 |
| Ligands | 115.25 |
| R.m.s. deviations |  |
| Bond lengths (Å) | 0.003 |
| Bond angles (°) | 0.578 |
|  |  |
| **Validation** |  |
| MolProbity score | 1.61 |
| Clashscore | 5.27 |
| Poor rotamers (%) | 1.52 |
| Ramachandran plot |  |
| Favored (%) | 96.88 |
| Allowed (%) | 3.00 |
| Disallowed (%) | 0.11 |
