# Supplementary material for: Structural and biochemical characterization of the key components of an auxin degradation operon from the rhizosphere bacterium Variovorax
Source: PLoS Biol. 2023 Jul 17;21(7):e3002189. doi: 10.1371/journal.pbio.3002189 (PMC10374108; doi:10.1371/journal.pbio.3002189)
Supplement: Attachment — Submitted filename: Response-to-Reviewers.docx [file pbio.3002189.s016.docx]

**Response to the Reviewers and Editors:**

REVIEWS:

***Reviewer #1:*** *The manuscript by Ma, et al. describes the ligand binding, structure and activity of proteins from the Variovorax auxin-degradation operon responsible for IAA binding and degradation. The authors performed crystallization studies and enzymatic assays to demonstrate IAA binding and enzymatic activity by AdgB, I, J and H. The degradation product of IAA catalysed by AdI/H/J was further characterized by mass spectrometry. The genes responsible for IAA degradation were then tested in E. coli and IAA degradation tested. Understanding the enzymes involved in IAA degradation and the crosstalk between plants and bacteria is of broad interest to the plant biology community.*
**Authors:** We thanks the reviewer for the positive comments.
*Certain points need to be addressed to better clarify the results of the manuscript and to put it into a broader context of what is known about IAA degrading bacteria-*
**Authors:** We sincerely appreciate the insightful comments and professional suggestions. According modifications have been made as follows.
*The results need to be discussed in context of recent literature, in particular Conway, et al. 2022* [*https://doi.org/10.1038/s41564-022-01244-3*](https://doi.org/10.1038/s41564-022-01244-3) *The catalytic mechanisms and different reaction products should be explained. As the nomenclature is different, it is difficult for the reader to follow. Are the authors using V. paradoxus in this study? Is this the same operon as described in Conway, et al.?*
**Authors:** We’re sorry for the confusion. We have studied the same locus, although using distinct methods in comparison to Conway et al. (Nature Microbiology, 2022). Genes in this locus were originally referred with gene IDs (Finkel et al., Nature, 2020), so we named them as auxin degradation genes (*adg*) in our study. In the revision, for clarity and consistency, we have renamed all the genes and proteins following the latest work published by Conway et al. (Nature Microbiology, 2022).

Although we have characterized IAA transformation in vitro, the catalytic mechanism elucidated in our study is consistent with the report by Conway et al through bacterial metabolic analysis (Nature Microbiology, 2022). They showed that the 2-hydroxyindole-3-acetic acid (minor tautomer) is an important intermediate in IAA degradation for the *iad* pathway, like the *iac* pathway. It should be noted that the 2-oxindole-3-acetic acid as an “enol” is chemically unstable, and will be converted into its stable "keto" form, 2-oxindole-3-acetic acid (oxIAA, major tautomer) (**please see below figure**, and also revised Figure 5E), consistent with our observation. Inside bacteria, however, further degradation occurs and these intermediates could be further degraded to anthranilic acid in the *iad* pathway. Further, consistent with Conway et al., we also identified AdgH/I/J (now named as IadC/D/E) as the key enzymes mediating the initial transformation of IAA. For clarity, according changes have been made in revision in **Results** and **Figures**, and we have discussed our study in the context of previous and the latest work.

*I am unclear as to the applications the authors envision for this operon in engineering the rhizosphere microbiome. This would be making transgenic soil bacteria to alter RGI?*
**Authors:** Sorry for the confusion. As stated in the *Introduction*, auxins control many essential cellular processes in plants such as cell division and differentiation, therefore the growth and yields. Bacterial regulation of auxin levels could also change these processes by altering the auxin pool (e.g., the root growth promoting capacity of *Variovorax*). Therefore, introduction of growth-promoting bacteria or optimization of the plant-associated bacterial community could potentially substantially promote the growth and yields of crops and reduce the use of fertilizers [1-5]. Engineering of growth-promoting bacteria have been performed to make the best use of the capacity [6]. Elucidation of the underlying mechanism of bacterial auxin regulation (e.g., the IAA inactivation mechanism by *Variovorax*) would guide the use and modulation of plant-associated bacteria (e.g., genetic engineering) in agriculture [2, 5, 6]. According modifications have been made in **Introduction** in revision.

*ITC measurements need s.d. or s.e.m. How many times were these measurements performed? For thermal shift assays in Fig. 1 the error bars are very faint and hard to see.*
**Authors:** We thank the reviewer for the suggestions. All the ITC measurements were performed at least three times independently. We have calculated the SD and revised the figures (revised **Figs 1C-D, 2D and 3A**). Color of error bars in revised **Figure 1E** have been modified accordingly.

*Given that AdgB is not in complex with IAA, docking studies were done. SAXS could be used to determine if AdgB adopts closed conformation upon IAA binding as co-crystallisation and soaking did not work.*
**Authors:** We appreciate the reviewer’s useful suggestion. In the revision, we have performed SAXS analysis of AdgB (now named as IadK2) in complex with IAA and confirmed the closed conformation (**revised supplementary Figure 2D**).

*Mutations of AdgA to bind IAA based on the structural and modelling studies of AdgB would be interesting to further support the model for IAA specific binding.*
**Authors:** Thanks for the suggestion. We have performed point mutations for substitute residues in the potential ligand binding pocket of AdgA (now named as IadK3) with corresponding amino acids of AdgB (now named as IadK2). Interestingly, none of the mutations could make IadK3 bind with IAA (**please see below figure**) and mutation of S137P caused precipitation of IadK3. One potential explanation could be that residues beyond the putative ligand binding pocket may regulate the conformation of IadK3 and ligand binding specificity. Further studies would be required to further examined the ligand binding specificity of IadK3.

*The structures in Fig.3 examine both X-ray and EM structures with the X-ray structure of AdgH/I in the apo form and the EM structure in the IAA bound form. A larger portion of the EM map should be shown around the active site and more detailed comparison of the conformation of the side chains in the apo and ligand bound form described. How well-positioned are the side chains in the EM model vs. the X-ray structure?*
**Authors:** We appreciate this useful suggestion. A new supplementary figure (**revised Figure S5**) has been included in the revision illustrating the electron densities around the active sites of both the X-ray and Cryo-EM structures, and a zoom-in structural comparison of the active sites. As shown in the figure, the side chains in both structures are well positioned.

*Fig. 4 -The schematic illustration is too small and uninterpretable. It is unclear where the electron is coming from and where it is going and how the reaction is catalysed.*
**Authors:** Thanks. Modifications have been made in revision. Figure 4E has been enlarged and the electron transport pathway are indicated in the figure.

*For the crystal tables, please provide CC1/2, Ramachandran statistics should be included. The clash scores and R factors are quite high for 7YLT. Is this structure fully refined? Also there seem to be a lot of RSTZ outliers for all structures.* **Authors:** CC1/2 and Ramachandran statistics have been included in the **revised supplementary Table 1**. The high clash score and R values were potentially related to the limited data quality in some high-resolution diffractions, which have been removed (the resolution is now reduced to 2.3 Å). The structure for 7YLT have been further refined with a final Rwork / Rfree values at 0.1864/0.2276.

*Supp Fig. 3 The SAXS experimental curve and theoretical model are very different. The fit looks extremely poor, especially at high q range, can the authors address this?*
**Authors:** We thank the reviewer for pointing out this issue. We agree with the reviewer that the fitting between the experimental data and the theoretical model was not good. Since we have the high-resolution cryo-EM structure resolved already for AdgH/AdgI (now named as IadD/IadE, Figure 3), we have removed this supplementary data in revision (**revised Fig S4**).

***Reviewer #2:*** *This manuscript is very straightforward, and summarizes an impressive amount of work, including the biochemical characterization of three different enzymes involved in IAA transport and metabolism. Overall, the claims are well supported. The findings are important, as they increase our understanding of how microbes can alter the concentration of the plant hormone IAA, an auxin, in plant roots and the rhizosphere.*
**Authors:** We truly appreciate the reviewer’s positive comments of our work.
*The manuscript is succinctly written, and in some places additional key information and details need to be provided so assist the readers in understanding the experiments and to strengthen the rationale for the work. Additionally, I believe use of the term "degradation"  is often inappropriately used, and thus confusing to the reader.*
**Authors:** We thank the reviewer for the suggestions. More experimental details were included in the revised results and methods, and the use of “degradation” are corrected with “transformation” or “conversion” etc. in the revision.

Scientific comments and concerns
1.       *An IAA degradation locus referred to as iad has been recently described (Conway et al, 2022 Nature Microbiology).  Is the adg locus discussed here distinct from that locus?*

**Authors:** We’re sorry for the confusion. We have studied the same locus, although using distinct methods in comparison to Conway et al. (Nature Microbiology, 2022 [7]). Genes in this locus were originally referred with gene IDs (Finkel et al., Nature, 2020 [1]), so we named them as auxin degradation genes (*adg*) in our study. In the revision, for clarity and consistency, we have renamed all the genes and proteins following the latest work published by Conway et al. (Nature Microbiology, 2022). Hope this will address the confusion.

2.      *The naturally occurring auxin phenyl acetic acid (PAA) should be included in the ITC experiments investigating substrate specificity. This is important to examine before the authors can claim that the protein has specificity for bicyclic compounds.*

**Authors:** We thank the reviewer for this kind suggestion. We have measured the binding of PAA to AdgA/B solute binding proteins (now named as IadK2/K3) and found no binding, which support our conclusion. This data has been included in the revision (**revised Fig S2**).

*3.      I was confused by the use of the term degradation, when the authors actually mean transformation (or conversion) of  IAA into oxIAA, which is presumably inactive, but still present in the cell.*

**Authors:** Yes, we agree the reviewer. All the use of “degradation” are corrected with “transformation” or “conversion” etc. in the revised manuscript.

*4.      It seems the authors believe that the SBP proteins are required for entry of IAA into the cell. However, at acidic pH (e.g. pH 5.8) much of the IAA is protonated, and thus may be able to diffuse across the membrane without the aid of transporters (at least this is what plant biologists who study IAA assume).  It would be very interesting to test this idea in a Varivorax adgB mutant.  This question may be beyond the scope of this manuscript, but could be addressed in future studies.*

**Authors:** Yes, we agree with the reviewer and believe this is a very interesting question. Although we did not study with *Varivorax* *adgB* (now named as *iadK2*) mutant strain, we explored using the engineered *E. coli*. Despite in neutral pH medium (re-measured and confirmed), expression of *iadK2* (WT or mutants) did not affect IAA degradation by the engineered *E. coli (****revised Figs6C, S11)***. However, further study using *Varivorax* *IadK2* mutants would be required in the future to clarify whether and how *iadK2* gene would contribute to IAA degradation by *Varivorax*.

*5.      The question about pH and entry of IAA into bacteria cells arose when I was considering the experiment shown in Fig. 6 (ecotpic expression of adg genes in E. coli). Presumably these experiments were done in pH7 media, but it would be helpful to confirm that the LB used in the IAA degradation assays was at pH 7 .*

**Authors:** Thanks for the suggestion. Yes, we have remeasured and confirmed that the culture medium was at neutral pH.

*6.      A control is missing for the experiment shown in Fig. 4 B. How can we ensure that the colormetric change does not occur spontaneously given enough time? A "no enzyme added" negative control is needed. Also, what is the time frame shown for the panel on the right, compared to the graph on the left?*

**Authors:** We appreciate the reviewer’s kind suggestions. We have re-performed the activity assays with the “no enzyme’ control (**revised Figure 4B**). The reaction time for the left panel have been indicated in the revised figure legend (revised Figure 4B, 30min).
*7.      More information regarding what is shown on the y axes in fig. 4 would be helpful.*
**Authors:** The axes tiles have been modified in revision.

*Editorial comments
Introduction:
1.      I believe the PAA, a natural and active form of auxin, is more abundant than IAA in many plants.*

**Authors:** We thank the reviewer for bringing out the information. Indeed, PAA was found more abundant than IAA in some plants. We have modified the introduction in revision to make it more precise and accurate.

*2.      Is reference 9 the appropriate citation for the general statement that "most rhizobacteria are found capable of producing IAA"? Referencing a review article would be more suitable.*

**Authors:** We thank the reviewer for the suggestion. We have revised the **reference** and cited a review article (Ref 7) as suggested.

*8.      An IAA degradation locus referred to as iad has been recently described (Conway et al, 2022 Nature Microbiology).  Are the adg genes here distinct from that locus?*

**Authors:** We’re sorry for the confusion. Yes, we have characterized the same operon as Conway et al. (Nature Microbiology, 2022). Since genes in this locus were originally referred with long gene IDs (Finkel et al., Nature, 2020), we named them as auxin degradation genes (*adg*) in our study. In the revision, we have renamed all the genes and proteins to be consistent with Conway et al. (Nature Microbiology, 2022). Hope this will address the confusion.

*9.      Is it appropriate to refer to conversion of IAA to oxIAA as degradation? I believe the term "inactivation" might be more appropriately used throughout the manuscript, until the discussion section, when a larger picture is provided.*

**Authors:** Thanks a lot for the advice. We have replaced “degradation” with ”transformation” in the revision. The use of “inactivation” have also been adjusted according to the suggestion.

*10.     Figure 5 legend:
a.      panel A- it would be helpful to indicate the color of the traces for before and after the transformation
b.      Panel c: what concentration of compounds are used in the experiment shown?*
**Authors:** Information has been added in the revision.

*Results
11.     Page 4, line the term "predicted" should be used when referring to products of genes that have not been characterized yet. The adg operon IS PREDICTED to encode….*
**Authors:** Change has been made accordingly in the revision.

*Methods:
12.     The Genbank accession numbers should be provided so that readers can analyze the genes/proteins*
**Authors:** Thanks for the suggestion. We have added the Gene IDs in the revised methods.


***Reviewer #3:*** *This study builds on an earlier report by Finkel et al., 2020, which defined an auxin (IAA) degradation locus in the bacterium Variovorax paradoxus. Here the authors define (1) the structure and auxin binding mechanism for the ABC transporter solute binding protein AdgB and (2) a structure of an AdgH/AdgI non-heme Rieske dioxygenase complex. The authors further demonstrate that AdgH/I/J can catalyze IAA degradation to oxIAA. Overall, this is a well designed/executed study that combines quantitative biochemistry, structural biology and enzymology to characterize a novel bacterial IAA degradation pathway. The crystal and cryoEM structure show good sterochemistry and refinement statistics. As a non-expert in this field, I had however some difficulties to compare this work to earlier studies. In addition, there are some minor technical issues worth considering. I will summarize my points below:*
**Authors:** We thank the reviewer for the comments. We are sorry for the confusion caused by the inconsistent gene names used in our study and the recent work done by Conway et al (Nature Microbiology, 2022). In the revision, we have renamed all the genes according to the earlier work.
*1. MAJOR: A recent study by Conway et al, 2022 (*[*https://doi.org/10.1038/s41564-022-01244-3*](https://doi.org/10.1038/s41564-022-01244-3)*) reported the gene products iadCDE as the minimal functional unit required for IAA degradation in Variovorax, and the regulation of root growth inhibition of a plant model (compare Fig. 1c in this study). Do the authors use the same nomenclature in this work (Fig. 1A) and if so, does that mean that there are two auxin degradation pathways encoded in Variovorax gene cluster, one represented by iad/adgCDE and one by adgH/I/J? If so, why are iadD and iadE necessary and sufficient for IAA degradation and root growth inhibition mediated by Variovorax (Conway et al, 2022, Fig. 1d)?*
**Authors:** We’re sorry for the confusion. We have studied the same locus, although using distinct methods in comparison to Conway et al. (Nature Microbiology, 2022). Genes in this locus were originally referred with long gene IDs (Finkel et al., Nature, 2020), so we named them as auxin degradation genes (*adg*) in our study. In the revision, for clarity and consistency, we have renamed all the genes and proteins following the latest work published by Conway et al. (Nature Microbiology, 2022) (revised Figure 1A and other figures, manuscript).

The complete dioxygenase system composed of the reductase (IadC/AdgJ) and all the dioxygenase subunits (IadD-IadE/AdgH-AdgI) would be required for the transformation of IAA, as demonstrated by our in vitro assays (Figure 4A). Inside bacteria, the function of IadC may potentially be partially compensated by other reductase proteins in the absence of IadC in some bacterial strains (e.g., *V. paradoxus* CL14 in Conway’s study), but the presence of IadC would significantly speed up IAA conversion (Fig. 1d in Nature Microbiology 2022), and is strictly required for other bacterial strains (e.g., *Arabidopsis* in Conway’s study and *E. coli* in our study). Hope this will address the confusion.

*2. MAJOR: I understand that iadB/adgB is the bacterial ABC transporter enabling IAA uptake into the cytoplasm of Variovorax. Assuming that all other degradation enzymes are present in the cytoplasm, does that mean that adgB is essential for both the iadC/D/E and the adgH/I/J pathways? Has this been experimentally tested, for example by genetic epistasis analysis?*
**Authors:** As explained above, *iadC/D/E* and the *adgH/I/J* are the same set of genes in the auxin degradation operon of *Variovorax*. Mutagenesis studies on the role *adgB* (now named as iadK2 to be consistent with the earlier study) have been performed in *E. coli* (**Figs 6B-C, S11**). The strong binding of AdgB/IadK2 with IAA is likely associated with the superior IAA degradation property of *Variovorax*. We agree with the reviewer that further mutagenesis studies of *adgB/IadK2* in *Variovrax* would be required to clarify its role in IAA degradation.

*3. MINOR: As anticipated, AdgB could engage IAA with nanomolar affinity, whereas no obvious binding was detected for AdgA and IAA. Why was it anticipated that AdgA would not bind IAA? This could be discussed later in the text, when the authors compare the degree of conservation of the putative IAA binding pocket in both proteins.*

**Authors:** Thanks for the suggestion. Sorry for the confusion. “As anticipated” was removed in the revision. We have modified the text accordingly.

*4. MAJOR: Fig. 1C, can the authors rationalize the bimodal binding behavior for recombinant AdgB in the ITC assay? The structure seems to indicate a single IAA binding site?*
**Authors:** Yes, indeed there is only one IAA binding site in AdgB (now named as IadK2 to be consistent with Nature Microbiology, 2022). The bimodal binding ITC profile of IAA to AdgB was most likely related to the induced closure of AdgB/IadK2 from the “open” conformation to the “closed” conformation. One possible explanation is that there could be an intermediate, IAA loosely bound state (close to the “open” conformation) coexisting with the tight, IAA-bound state (“closed” conformation) in solution.

*5. MAJOR: Conway et al, 2022 et al reported iad/C/D/E to form an IAA degradation complex. Have these proteins been tested in the ITC assays shown in Supplementary Fig. 1, and if so, why was no binding detected? It seems that MarR domains,  that show high affinity IAA binding (Kd ~0.3 uM) (Conway et al. 2022) were excluded from the analysis in Fig. S1? This should be mentioned in the figure legend.*
**Authors:** Please reference to above explanation in point1 and 2. Consistent with Conway et al., we also found that AdgH/I/J (now named as IadC/D/E as well for consistency) form the dioxygenase system, which bind and transform IAA (Figure 3a, 4, 6 and figure S1). AdgI/IadD is the catalytic subunit, which directly associate with the substrate IAA. The repressor gene *MarR* was not included in our study and we made this information clear in the **revised figure S1**. The gene names and figure legends have also been revised for better clarity as suggested.

*6. MAJOR: In the Figure 5, the authors nicely demonstrate that AdgH/I/J can catalyze IAA oxidation. Can these data be integrated with the metabolic analysis by Conway et al, 2022, Fig. 4? Could this imply that iadCDE and adgHIJ are part of one degradation pathway, and that adgHIJ may use 2-hydroxy-indole-3-acetic acid or dioxindole-3-acetic acid (DOAA) as substrates in vivo? Please clarify.*

**Authors:** We sincerely thank the reviewer’s valuable comments. Our result is consistent with the metabolic analysis of Conway et al. (Nature Microbiology, 2022), although we have characterized IAA transformation in vitro. They showed that the 2-hydroxyindole-3-acetic acid (minor tautomer) is an important intermediate in IAA degradation for the *iad* pathway, like the *iac* pathway. It should be noted that the 2-oxindole-3-acetic acid as an “enol” is chemically unstable, and will be converted into its stable "keto" form, 2-oxindole-3-acetic acid (oxIAA, major tautomer) (**please see below figure**, and also revised Figure 5E), consistent with our observation. Inside bacteria, however, further degradation occurs and these intermediates could be further degraded to anthranilic acid in the *iad* pathway. Further, consistent with Conway et al., we also identified AdgH/I/J (now named as IadC/D/E) as the key enzymes mediating the initial transformation of IAA. For clarity, according changes have been made in revision in **Results** and **Figures**, and we have discussed our study in the context of previous and the latest work.

*7. MINOR: Figure 5a/Figure S7. Could the HPLC buffer system or column be optimized to obtain a sharper peak for IAA?*
**Authors:** We appreciate the suggestion. We have attempted to further optimize the separation of the IAA deactivation mixture or IAA using the HPLC with distinct gradients or a new column (**please refer to the figure below** showing the separation with distinct solvents or gradients). However, these did not substantially improve the separation of peaks. But even so, as shown in **Figure 5A and Figure S9**, we have included all the required controls to support and validate our conclusion on the IadC/D/E(AdgH/I/J)-mediated transformation of IAA to oxIAA. Hope this will address the concern.

*8. MAJOR: Fig. 5. I would recommend to determine rate constants for the different substrates.*
**Authors:** Thanks for the suggestion. In this study, we have utilized the Salkowski reagent to quantify and monitor the change of IAA during treatment with the IadC/D/E(AdgH/I/J) dioxygenase system, which develop a pink color and adsorption at 530 nm after mixing with IAA. This method has a relatively narrow linear range (0-0.25 mM). Determination of the enzymatic rate constants however would require a larger linear concentration range to precisely quantify the amounts of transformed IAA (**please see the figure below**). Therefore, we have instead measured the time kinetics and enzyme concentration dependence with a substrate concentration of 0.1 mM (**revised Figure 4B**). Hope the reviewer could understand this challenge.

*9. MINOR: Fig. 6D may need to be revised depending on the outcome of the analysis on point 6 (see above)*

**Authors:** We appreciate this useful suggestion. We have modified Figure 6D as explained in point 6 (please see above).

*10. MINOR: Introduction: "Elucidating the underlying mechanism of bacterial IAA degradation would help to maximize the beneficial effects of auxin degradation for ecological agriculture." How would that be achieved?*
**Authors:** Sorry for the confusion. As stated in the *Introduction*, auxins control many essential cellular processes in plants such as cell division and differentiation, therefore the growth and yields. Bacterial regulation of auxin levels could also change these processes by altering the auxin pool (e.g., the root growth promoting capacity of *Variovorax*). Therefore, introduction of growth-promoting bacteria or optimization of the plant-associated bacterial community could potentially substantially promote the growth and yields of crops and reduce the use of fertilizers [1-5]. Engineering of growth-promoting bacteria have been performed to make the best use of the capacity [6]. Elucidation of the underlying mechanism of bacterial auxin regulation (e.g., the IAA inactivation mechanism by *Variovorax*) would guide the use and modulation of plant-associated bacteria (e.g., genetic engineering) in agriculture [2, 5, 6].

*11. MINOR: Introduction: "Dissecting and defining the functional mechanism of the adg operon is therefore of significant importance for the applications in ecological and stress agricultures." What type of application exist that make use of bacterial IAA degradation, please specify.*
**Authors:** Please refer to above response to point 10.

***COMMENTS FROM THE ACADEMIC EDITOR***

*I agree with the reviewers that the lack of comparison the Conway is a major weakness and it isn’t clear where the “adg” nomenclature/operon (instead “iac” in the Finkle 2020 paper and iac/iad in the Conway 2022 paper) came from. Similarly, the operon structures in Conway seems a bit different than in this paper so I’m wondering if these are distinct operons. However, if that’s the case, then the authors of this paper should clearly state how they decided to study the adg operon and it’s demonstrated role in auxin degradation (and if it is different from or the same as iac or iad).*
**Authors:** Thanks for the comments and sorry for the confusion. We have characterized the same operon as Conway et al. (Nature Microbiology, 2022). Since genes in this locus were originally referred with long gene IDs (Finkel et al., Nature, 2020), we named them as auxin degradation genes (*adg*) in our study. In the revision, we have renamed all the genes and proteins to be consistent with Conway et al. (Nature Microbiology, 2022). According modifications have been made in revision to address such confusion.

*Futhermore, the IAA degradation reactions described are different. The Conway paper describes that the iac operon breaks down IAA through DOAA to catechol while the iad locus converts IAA to anthranilic acid. In this paper, they describe oxIAA as the next step in degradation by the adg degrading operon. Either Ma et al. are describing the same operon but found a different reaction and degradation product or they’re describing a distinct operon suggesting 3 Variovorax degrading pathways. Importantly, I couldn’t find the species or strain listed anywhere in the materials and methods so it’s not clear if this is the same strain. This definitely needs to be clarified and either outcome (two mechanisms, or inconsistency with previous papers) should be clarified.*

**Authors:** Sorry for the confusion. Our result is consistent with the metabolic analysis of Conway et al. (Nature Microbiology, 2022). Please refer to our response to **Reviewer #3 point 6**. Species, strains, and gene IDs have been included in the revised method. We have renamed all the genes and proteins to be consistent with Conway et al. (Nature Microbiology, 2022). Hope this will address the concern.

**Reference:**

1.	Finkel OM, Salas-Gonzalez I, Castrillo G, Conway JM, Law TF, Teixeira P, et al. A single bacterial genus maintains root growth in a complex microbiome. Nature. 2020;587(7832):103-8. Epub 20200930. doi: 10.1038/s41586-020-2778-7. PubMed PMID: 32999461.

2.	Baudoin E, Lerner A, Mirza MS, El Zemrany H, Prigent-Combaret C, Jurkevich E, et al. Effects of Azospirillum brasilense with genetically modified auxin biosynthesis gene ipdC upon the diversity of the indigenous microbiota of the wheat rhizosphere. Res Microbiol. 2010;161(3):219-26. Epub 20100204. doi: 10.1016/j.resmic.2010.01.005. PubMed PMID: 20138146.

3.	Chaiharn M, Lumyong S. Screening and optimization of indole-3-acetic acid production and phosphate solubilization from rhizobacteria aimed at improving plant growth. Curr Microbiol. 2011;62(1):173-81. Epub 20100616. doi: 10.1007/s00284-010-9674-6. PubMed PMID: 20552360.

4.	Bharucha U, Patel K, Trivedi UB. Optimization of Indole Acetic Acid Production by Pseudomonas putida UB1 and its Effect as Plant Growth-Promoting Rhizobacteria on Mustard (Brassica nigra). Agricultural Research. 2013;2(3):215-21. doi: 10.1007/s40003-013-0065-7.

5.	Zuniga A, Poupin MJ, Donoso R, Ledger T, Guiliani N, Gutierrez RA, et al. Quorum sensing and indole-3-acetic acid degradation play a role in colonization and plant growth promotion of Arabidopsis thaliana by Burkholderia phytofirmans PsJN. Mol Plant Microbe Interact. 2013;26(5):546-53. doi: 10.1094/MPMI-10-12-0241-R. PubMed PMID: 23301615.

6.	Beyeler M, Keel C, Michaux P, Haas D. Enhanced production of indole-3-acetic acid by a genetically modified strain of Pseudomonas fluorescens CHA0 affects root growth of cucumber, but does not improve protection of the plant against Pythium root rot. FEMS Microbiology Ecology. 1999;28(3):225-33.

7.	Conway JM, Walton WG, Salas-Gonzalez I, Law TF, Lindberg CA, Crook LE, et al. Diverse MarR bacterial regulators of auxin catabolism in the plant microbiome. Nat Microbiol. 2022;7(11):1817-33. Epub 20221020. doi: 10.1038/s41564-022-01244-3. PubMed PMID: 36266335; PubMed Central PMCID: PMCPMC9613470.
