# Supplementary material for: Structural and biochemical characterization of the key components of an auxin degradation operon from the rhizosphere bacterium Variovorax
Source: PLoS Biol. 2023 Jul 17;21(7):e3002189. doi: 10.1371/journal.pbio.3002189 (PMC10374108; doi:10.1371/journal.pbio.3002189)
Supplement: Attachment — Submitted filename: Responses.docx [file pbio.3002189.s017.docx]

**Response to the Editors:**

Thank you for your patience while we considered your revised manuscript "Molecular Mechanism for Bacterial Degradation of Plant Hormone Auxin" for publication as a Research Article at PLOS Biology. This revised version of your manuscript has been evaluated by the PLOS Biology editors and the Academic Editor.

Based on our Academic Editor's assessment of your revision, I am pleased to say that we are likely to accept this manuscript for publication, provided you satisfactorily address the following data and other policy-related requests that I have provided below (A-F):

(A) We would like to suggest the following modification to the title:

“Structural and biochemical characterization of the key components of an auxin-degradation operon from the rhizosphere bacterium Variovorax”

***Authors:*** *Thanks for the suggestions. We have revised the manuscript title to “Structural and biochemical characterization of the key components of an auxin-degradation operon from the rhizosphere bacterium Variovorax” as suggested.*

(B) You may be aware of the PLOS Data Policy, which requires that all data be made available without restriction: http://journals.plos.org/plosbiology/s/data-availability. For more information, please also see this editorial: http://dx.doi.org/10.1371/journal.pbio.1001797

Note that we do not require all raw data. Rather, we ask that all individual quantitative observations that underlie the data summarized in the figures and results of your paper be made available in one of the following forms:

- Supplementary files (e.g., excel). Please ensure that all data files are uploaded as 'Supporting Information' and are invariably referred to (in the manuscript, figure legends, and the Description field when uploading your files) using the following format verbatim: S1 Data, S2 Data, etc. Multiple panels of a single or even several figures can be included as multiple sheets in one excel file that is saved using exactly the following convention: S1_Data.xlsx (using an underscore).

- Deposition in a publicly available repository. Please also provide the accession code or a reviewer link so that we may view your data before publication.

Regardless of the method selected, please ensure that you provide the individual numerical values that underlie the summary data displayed in the following figure panels as they are essential for readers to assess your analysis and to reproduce it:

Figure 1C-E, 2D-E, 3A, 3F, 4A-B, 4D, 4F, 5A-B, 6B-C, S1B-E, S2A-B, S3D, S4A, S8B, S11

NOTE: the numerical data provided should include all replicates AND the way in which the plotted mean and errors were derived (it should not present only the mean/average values).

***Authors:*** *Source data and uncropped gel image have been included in* ***S1 Data*** *and* ***S1 Raw image*** *supporting information files.*

(C) Thank you for depositing the structural data in the PDB (7YLT, 7YLS, 7YLR, 8H2T) and EMDB databases (EMD-34443). However, I note that the data is currently on hold. We ask that you please make this data publicly available before publication.

***Authors:*** *We have submitted the release requests to PDB for PDB entries (7YLT, 7YLS, 7YLR, 8H2T) and EMD-34443. These entries are scheduled to be released on June 14, 2023.*

(D) Please also ensure that each of the relevant figure legends in your manuscript include information on *WHERE THE UNDERLYING DATA CAN BE FOUND*, and ensure your supplemental data file/s has a legend.

***Authors:*** *Relevant source data information has been indicated in the figure legends in the revision. Titles have been added for all the supplemental data, figures, and tables.*

(E) We require the original, uncropped and minimally adjusted images supporting all blot and gel results reported in the following Figures:

Figure S4B

We will require these files before a manuscript can be accepted so please prepare and upload them now. Please carefully read our guidelines for how to prepare and upload this data: https://journals.plos.org/plosbiology/s/figures#loc-blot-and-gel-reporting-requirements

***Authors:*** *Uncropped gel image for Figure S4B have been provided in the supporting information file* ***S1 Raw image****.*

(F) Please ensure that your Data Statement in the submission system accurately describes where your data can be found and is in final format, as it will be published as written there. This includes referencing where the underlying data can be found in the Supplementary Information.

***Authors:*** *Data Availability statement has been confirmed and revised with the source data statement.*

-----------------------------------------

As you address these items, please take this last chance to review your reference list to ensure that it is complete and correct. If you have cited papers that have been retracted, please include the rationale for doing so in the manuscript text, or remove these references and replace them with relevant current references. Any changes to the reference list should be mentioned in the cover letter that accompanies your revised manuscript.

***Authors:*** *We have confirmed the reference list in this manuscript.*
